# Quality of Life in Hungarian Parents of Autistic Individuals

**DOI:** 10.1007/s10803-024-06243-3

**Published:** 2024-01-27

**Authors:** Marta Volgyesi-Molnar, Miklos Gyori, Valsamma Eapen, Zsofia Borsos, Agnes Havasi, Zoltan Jakab, Laszlone Janoch, Vivien Nemeth, Tamasne Oszi, Agota Szekeres, Krisztina Stefanik

**Affiliations:** 1 Hungarian Academy of Sciences - ELTE University ’Autism in Education’ Research Group, Budapest, Hungary; 2 Faculty of Special Education, Institute of Special Needs Education for People with Atypical Behaviour and Cognition, ELTE University, Budapest, Hungary; 3 Discipline of Psychiatry and Mental Health, School of Clinical Medicine, Faculty of Medicine and Health, University of New South Wales, Sydney, NSW Australia; 4 Academic Unit of Infant, Child and Adolescent Psychiatry Services (AUCS), South Western Sydney Local Health District & Ingham Institute, Liverpool, Australia; 5 Faculty of Special Education, Institute for the Psychology of Special Needs, ELTE University, Budapest, Hungary; 6 Faculty of Education and Psychology, Doctoral School of Education, ELTE University, Budapest, Hungary; 7 Hungarian University of Agriculture and Life Sciences, Institute of Education, Kaposvar, Hungary

**Keywords:** Autism spectrum disorders, Parents, Quality of life, Psychological well-being, Intervention

## Abstract

**Purpose:**

Parents of autistic individuals have been known to have a lower overall quality of life (QQL) than those of typically developing children. We present the first Hungarian large-sample study whose objective was to explore the differences in QOL between parents of autistic individuals (AS) and those of neurotypical (NT) persons.

**Methods:**

Based on the ABCX model we developed a questionnaire comprising standardized scales to characterize the life of parents involved. Our data came from parents of 842 individuals (ASD = 521, NT = 321) between 0 and 49 years. Battery deployed standardized instruments to examine quality of life (WHO-QQL BREF and Quality of Life in Autism questionnaire, QOLA). We assessed the families’ socio-economic/demographic characteristics, parents’ psychological well-being, the autistic/neurotypical individuals’ characteristics, and the interventions.

**Results:**

Our data showed significantly lower QOL in parents of autistic individuals in all domains of questionnaires. We analyzed 20 relevant factors to uncover the predictors of parental QOL. We confirmed the existence of most but not all predictors present in earlier literature and identified intervention-related predictors.

**Conclusion:**

Our study confirms the importance of supporting parents in their role, and of providing health and social supports that focus on quality of life, in addition to child care.

## Introduction

Families are often the major source of support for autistic people throughout much of their life (Lord et al., 2020). Raising a child with a diagnosis of autism spectrum disorder (ASD) affects parental quality of life negatively, compared to neurotypical children and children with chronic illness (Vasilopoulou & Nisbet, 2016; Pisula & Porębowicz-Dörsmann, 2017; Dey et al., 2019; Ni’matuzahroh, Suen et al., 2022). Parents of atypically developing children including ASD have their quality of life influenced by a complex system of factors: socio-demographic, socio-ecological, psycho-social factors, and those related to their child’s special condition (Isa et al., 2016; Ooi et al., 2016). Factors affecting quality of life also interact with coping strategies, stress, sense of coherence, and relationship between parents (Vernhet et al., 2019).

It is not completely known for each of these factors how they influence parental quality of life in the context of ASD (Eapen & Guan, 2016; Vasilopoulou & Nisbet, 2016; Sonido et al., 2020; Wang et al., 2022; Eapen et al., 2022).

### Conceptual Framework

Systemic models of quality of life help to obtain a picture of the strengths, needs, and difficulties of the individuals in focus, and their family members. This in turn serves as a basis in planning care and support (Summers et al., 2005). The *Double ABCX model* aims to capture a range of risk and protective factors as well as the phase of adjustment that family members experience as a stressful event unfolds (McCubbin & Patterson, 1983; Bohadana et al., 2019).

Numerous authors in the field of ASD have employed the ABCX model to measure family-level quality of life and stress levels (Stuart & McGrew, 2009; Manning et al., 2011; Pozo et al., 2014; Čolić et al., 2019). The model organises the factors involved in family adaptation based on the following four components: stressor severity (A; e.g., severity of the child’s symptoms), accumulating demands and additional stressors (aA, e.g., financial problems, illness, divorce); family’s internal resources (B; e.g., self-efficacy; locus of control); family’s external resources (bB; e.g., social support, reliable finances); family appraisal of the situation (C; e.g., challenge appraisal); coping strategies used (cC; e.g., problem-focused, emotion-focused), and outcome (xX, e.g., family quality of life) (McCubbin & Patterson, 1983; Čolić et al., 2019). In line with the *Double ABCX model*, it is worth reviewing the relevant factors (socio-demographic/economic, parental wellbeing and characteristics of the children) that are known to influence parental quality of life in autism spectrum disorder.

### Parental Quality of Life, Socio-Demographic and Socio-Economic Condition

In ASD, socio-economic factors affect parental quality of life from an early age of the child (Mathew et al., 2019); some such factors exert their influence in a largely uniform way on both parents. A substantial proportion of parents report *financial difficulties* accompanied by poorer quality of life, depression, and burnout (Hoefman et al., 2014). Dissatisfaction with family income, especially in the case of mothers, affects quality of life in a direct way (Dardas & Ahmad, 2014), while satisfaction in this regard is protective, and has a positive effect starting early on in the child’s life (Mello et al., 2019). It is well-known that parents of autistic children endure significant financial and employment burden associated with their children’s numerous service needs (Montes & Halterman, 2008; Wallace-Watkin et al., 2022). Regarding *family status* the existing results are somewhat controversial, for example, lower (Freedman et al., 2012) and higher (Hartley et al., 2010) rates of separation and divorce have both been found (Sonido et al., 2020). It seems that quality of the *relationship between parents*, is a crucial factor for quality of life and coping (Sonido et al., 2020; Wang et al., 2022). Based on previous literature number of *siblings* has been shown to exhibit a significant positive correlation with quality of life, and in this regard, more siblings (Dardas & Ahmad, 2014; Eapen & Guan, 2016; Sonido et al., 2020) and having non-autistic siblings (Eapen et al., 2022) have been found to predict better parental quality of life.

### Parental Quality of Life, and Psycho-Social Characteristics

Parents of autistic children experience higher levels of *stress* than found in other developmental conditions (Pisula & Porębowicz-Dörsmann, 2017; Ni’matuzahroh, Suen et al., 2022). Higher stress levels are related to the child’s difficult behavioural pattern (Craig et al., 2016), altogether negatively affecting quality of life for both parents (Dardas & Ahmad, 2014; Tung et al., 2014). According to the findings of an Australian study, higher scores in the positive dimension of self-compassion are correlated with better quality of life, whereas higher scores in its negative dimension were associated with higher stress levels (Bohadana et al., 2019). The relationship between the occurrence of depressive symptoms and autism spectrum disorder in a family is based on a complex interplay of genetic and environmental issues (Daniels et al., 2008), with some studies indicating the presence of depressive symptoms even before the birth of the autistic child while other studies have indicated that stress levels in the family due to having an autistic child may result in secondary *depressive symptoms* (Zablotsky et al., 2013; Wei et al., 2015). The frequency of depressive symptoms in these families is substantially higher than in families with typically developing children (Schnabel et al., 2020; Bispo-Torres et al., 2023). Low levels of control experienced by parents has been correlated with lower maternal, and to some extent, paternal, quality of life (Yamada et al., 2012). Likewise *competence*, *control*, and *coherence* are strongly correlated with the well-being of parents of autistic children (Frantzen et al., 2016; Ismail et al., 2022). Of these, parental sense of competence appears to be the most important construct (Frantzen et al., 2016) which is related to socio-economic status, the child’s symptoms, mood disturbances, and social support, although mothers and fathers exhibit a different pattern in this regard (Mathew et al., 2019). A particularly important protective factor that higher *self-esteem* (Lee et al., 2012), better sense of coherence (Eapen & Guan, 2016) are accompanied by better quality of life for parents of autistic children. In addition sufficient levels of *social support* available for parents positively influences well-being and quality of life, for both parents (Pozo et al., 2014; Schiller et al., 2021). Interestingly, whereas informal social support has a proven connection with quality of life, the relationship between formal support and quality of life is less well understood (Eapen & Guan, 2016; Vasilopoulou & Nisbet, 2016; Marsack & Samuel, 2017; Salomone et al., 2018; Sonido et al., 2020). Within the realm of informal social support (Ismail et al., 2022), the positive effects of neighbor support have been demonstrated by some studies in recent years (Hsiao, 2016; Mathew et al., 2019).

### Parental Quality of Life, and Factors Related to Children and Their Conditions

In addition to environmental and psychosocial factors, parental quality of life is also influenced by the child’s characteristics (Ooi et al., 2016). Initially it is the parents’ reaction to the diagnosis that crucially influences parental quality of life (Eapen & Guan, 2016). Some data suggest that in earlier years parents’ stress levels are elevated in ASD (Barker et al., 2011); however, other studies did not find a correlation between the *child’s age*, and parental stress, (Peters-Scheffer et al., 2012). In addition, it seems that transitions (Lounds et al., 2007), and accumulating stressors with age affect parental well-being at least as much as difficulties in early years (Smith et al., 2008). All by itself the level of *intellectual ability* does not directly influence parental well-being, according to some studies (Peters-Scheffer et al., 2012; Vernhet et al., 2022), however, in others it seems to be a protective factor for parental quality of life (Baghdadli et al., 2014). In this regard, level of adaptive functioning and behavioural profile have been found to impact parental quality of life more strongly than the child’s intellectual or cognitive capacity (Eapen et al., 2022). Further, *severity of autism-related symptoms* (Zablotsky et al., 2013; Pozo et al., 2014.; Eapen et al., 2022), *challenging behaviours* (Dardas & Ahmad, 2014; Tung et al., 2014), and child-related difficulties have been shown to negatively influence parental well-being and quality of life while also exacerbating the level of stress experienced by the parent (Walsh et al., 2013; Eapen & Guan, 2016; Salomone et al., 2018). It is well known that the presence of *associated problems* (Zablotsky et al., 2013), and high support needs are also associated with lower scores of maternal, and in part, paternal quality of life indicators (Yamada et al., 2012). It is a particulary important factor in relation to services that children’s *insitutional care* also influences parental quality of life (Eapen & Guan, 2016; Frantzen et al., 2016). If the child receives institutional care and parents can be involved, they report better quality of life (Musetti et al., 2021). Within this group the quality of life of those parents whose children receive inclusive education, or whose relationship with the professionals is better, tend to have more favorable quality of life (Alhazmi et al., 2018; Balcells-Balcells et al., 2019). Factors discussed so far influencing quality of life included in our study are shown in Table 1.

**Table 1:** Factors influencing quality of life included in our study

Parental Quality Of Life &		
**Socio-Demographic/Socio-Economic Condition**	**Psycho-Social Characteristics/Well-Being**	**Factors Related to Children and Their Conditions**
Dis/satisfaction with family income (-/+) (Hoefman et al., 2014; Dardas & Ahmad, 2014; Mello et al., 2019)	Higher stress levels (-) (Dardas & Ahmad, 2014; Tung et al., 2014)	Parents’ reaction to the diagnosis (+/-) (Eapen & Guan, 2016)
Costs associated with special care (-) (Montes & Halterman, 2008; Wallace-Watkin et al., 2022)	Positive/negative dimension of self-compassion (+/-) (Bohadana et al., 2019)	Child’s age (?) (Lounds et al., 2007; Smith et al., 2008; Barker et al., 2011; (Peters-Scheffer et al., 2012)
Family status (no divorce) (+/-) (Freedman et al., 2012; Hartley et al., 2010; Sonido et al., 2020)	Self-esteem (+) (Lee et al., 2012)	Level of intellectual ability (0/+) (Peters-Scheffer et al., 2012; Baghdadli et al., 2014; Vernhet et al., 2022)
Relationship between parents (+/-) (Sonido et al., 2020; Wang et al., 2022)	Competence and coherence (+) (Frantzen et al., 2016; Eapen & Guan, 2016; Ismail et al., 2022)	Better adaptive functioning and behavioural profile (+) (Eapen et al., 2022)
Siblings: more (+) (Dardas & Ahmad, 2014; Eapen & Guan, 2016; Sonido et al., 2020) Having non-autistic siblings (+) (Eapen et al., 2022)	Low levels of control (-) (Yamada et al., 2012)	Severity of autism-related symptoms, more challenging behaviours (-) (Zablotsky et al., 2013; Pozo et al., 2014.; Dardas & Ahmad, 2014; Tung et al., 2014; Eapen et al., 2022)
	Depression (-) (Zablotsky et al., 2013; Wei et al., 2015)	Presence of associated problems (-) (Zablotsky et al., 2013)
	Avaliable informal social support (e.g. neighbor) (+) (Pozo et al., 2014; Hsiao, 2016; Mathew et al., 2019; Schiller et al., 2021)	Higher support needs (-) (Yamada et al., 2012)
	Formal social support (?) (Eapen & Guan, 2016; Vasilopoulou & Nisbet, 2016; Marsack & Samuel, 2017; Salomone et al., 2018; Sonido et al., 2020)	Child’s insitutional care – better satisfaction/involvement/professional inclusion (+) (Eapen & Guan, 2016; Frantzen et al., 2016; Alhazmi et al., 2018; Balcells-Balcells et al., 2019; Musetti et al., 2021)

### The Picture in Central-Eastern Europe: Quality of Life of Parents

So far few studies have addressed the quality of life, psyhological well-being, and coping strategies of parents of autistic children in Central-Eastern Europe (Benjak, 2011; Pisula & Porębowicz-Dörsmann, 2017; Čolić et al., 2019). The regional overview by Čolić et al. (2019) examined 9 of the 17 countries in the region, and found that factors influencing parental quality of life resemble those identified in Western countries. Results from the East-European region indicate that parents in the ASD group lag behind parents of neurotypical children in terms of psychological well-being (coping strategies; stress), and the contributory factors included child-related challenges, satisfaction with family income, and access to support services. However, currently it is unclear how the magnitude of the difference, and the precise pattern of determinants differs from what is found in Western countries (Čolić et al., 2019).

### Context of the Present Research

Hungary is one of those countries in Central-Eastern Europe which, more than 30 years after the end of ’communism’, is economically a middle-income country with a unique political situation, and pattern of values (The Trading Economics Application Programming Interface, 2022). The cultural map is dominated, in a stable fashion, by individualist, secular-rational, and survival values (The Inglehart-Welzel World Cultural Map, 2020). Hungarostudy, a national representative health survey assessing the population’s physical and mental well-being, presented an unfavourable picture. Low life expectancy, unsatisfactory levels of quality of life, and poor psychological well-being of Hungarian people are underpinned by factors such as a dominant attitude of learned helplessness, the lack of a safe and predictable vision of the future, a marked lack of social capital (especially trust), and financial inequalities (Kopp & Kovács, 2006; Kopp & Martos, 2011). Still Hungary shows a promising picture in its system of autism care with important history, as modern, evidence-based professional care was brought into action in the 1980s (Balázs, 2004). In this continually evolving professional environment (involving legislative background, professional guiding principles, and education of evidence-based practices), however, a nationwide study covering the entire autism spectrum, broad age ranges, and involving family members, is still awaited. A few previous studies in Hungary have addressed some aspects of quality of life, although they did not cover the entire spectrum of autism (Bernát et al., 2017) and did not include a comprehensive parental quality of life study (Petri & Vályi, 2009). An international study covering seven countries (including our recent data), found that intercountry differences exist at both an overarching and domain level, which in turn indicates that the way in which overall ratings of how various aspects of ASD are perceived come together in much the same way, regardless of which cultural context people come from. In this analysis, Hungary places little emphasis on rights and personal development.

### Aims

It was the scarcity of data on ASD and quality of life that motivated the research group to conduct a large-sample, cross-sectional study of quality of life (QOL) and well-being (WB) comparing parents with autistic individuals with parents raising neurotypical individuals as a control group. Data collection took place in 2017-18. Our hypotheses were: (1) Similarly to international trends, quality of life and psychological well-being of Hungarian parents of autistic individuals will be significantly worse than those of parents raising neurotypical individuals. (2) Within the group of parents of autistic individuals, quality of life will be strongly related to certain factors; in particular the autistic individual’s symptoms, satisfaction with the autistic individual’s professional care, and parental well-being. We also asked whether there exist factors determining quality of life specific to parents of autistic individuals, that is, not applicable to neurotypical parents (and vice versa).

## Methods

### Research Design and Recruitment Procedures

Recruitment of participants and data collection took place on online platforms, with the help of the Hungarian Autistic Society and regional experts of our research group. Dimensions to characterize the sample were socio-economic status (parental education level), place of residence (area of residence types of all Hungarian regions were included), child’s age (from early years to adults), and level of functioning (the latter including intellectual ability and language skills). We ensured inclusion of participants from all combinations of values along these dimensions. The battery of questionnaires was filled out on a voluntary basis, and anonymously. To this end we developed an online platform, however, the battery was also available offline (paper version), in order not to miss parents with limited internet access for any reason. Ethical approval for the study was obtained from the Committee of Research Ethics of Bárczi Gusztáv Faculty of Special Education, Eötvös Loránd University (permission number: KEB/2017/003).

### Participants, Description of Sample

All questionnaires with group membership data (autism spectrum disorder AS or neurotypical NT) were evaluated. In the AS group, parents supplied data concerning their autistic child – in cases of more than one child being affected, the system made a random choice asking for data of one child in particular. In the control group a similar random choice was made. Parental statement served as evidence for the ASD diagnosis. The total number of questionnaires completed in the AS group was *N* = 521; (438 that is 84.1% female). Mean age was 42.4 (SD ± 8.3) years. Of participants’ children in the AS group 434 (83.3%) were male; the median age of individuals was 10.1 (IQR: 7.46, range: 2.2 to 47.2). The control group comprised 321 parents (289 that is 90% female); mean age was 42.4 (SD ± 8.7) years. Of their neurotypical children 153 (47.6%) were female; the median age in NT group was 10.7 years; (IQR: 12.3 range: 0.1 to 48.5). A summary of the demographic data including the participants’ children functional level is shown in Table 2.

**Table 2:** Characteristics of the sample (AS and NT groups)

	AS parental group (n = 521)	NT parental group (n = 321)
Sex % (n)	15.5 (81) male, 84.1 (438) female	10.0 (32) male, 90.0 (289) female
Age (years)	42.4 (SD ± 8.3)	42.4 (SD ± 8.7)
Family status % (n)		
Married/in relation	77.4 (401)	82.2 (264)
Single/divorced	20.9 (108)	14.3 (46)
Widow	1.7 (9)	3.4 (11)
Education level % (n)		
Elementary school or vocational high school without baccalaureat	13.4 (69)	3.5 (11)
High school with baccalaureat	34.9 (181)	17.1 (55)
Higher education [college or university]	51.7 (268)	79.4 (255)
Area of residence type % (n)		
Farmstead	1.2 (6)	0.3 (1)
Village	18.1 (94)	18.4 (59)
Township	43.2 (224)	32.7 (105)
County seat	21.0 (109)	25.5 (82)
Capital	16.6 (86)	23.1 (74)
Estimated relative monthly income % (n)		
Very low	7.1 (37)	1.2 (4)
Low	21.4 (111)	10.0 (32)
Average	48.5 (251)	54.2 (174)
Moderately above average	20.1 (104)	32.1 (103)
Substantially above average	2.9 (15)	2.5 (8)
Average age of autistic/neurotypical individuals years median (min/max)	10.1 years; (2.2/47.2)	10.7 years; (0.1/48.5)
Sex of the autistic/neurotypical individuals % (n)	83.3 (434) male, 16.7 (87) female	52.3 (168) male, 47.6 (153) female
Funcional level of individuals % (n)		
Low-functioning (LF)	12.1 (63)	0.3 (1)
Medium-functioning (MF)	20.9 (109)	1.2 (4)
High-functioning (HF)	51.4 (268)	90.1 (292)
Not classified	15.5 (81)	8.3 (27)

### Measures and Tools

Based on the ABCX model we developed a questionnaire comprising standardized scales and additional question blocks to characterize the life of parents involved. We assessed the families’ socio-economic and demographic situation; the parents’ social support; information concerning the child’s ASD traits and associated conditions; their strengths and weaknesses; finally, their education and intervention. Further, our battery deployed standardized instruments to examine quality of life, psychological well-being (depression, anxiety, stress, sense of coherence, coping, and parental sense of competence).

The *WHOQOL-BREF* is a brief version of WHOQOL-100. It has a Hungarian version, consisting of 26 items that examine four generic quality-of-life domains (physical health; psychological health; social relations, and environment); using these, and two additional items (rating one’s quality of life; satisfaction with one’s health) it constructs a quality-of-life profile (The WHOQOL Group, 1998; Paulik et al., 2007). Our group translated and used the *Quality of Life in Autism Questionnaire* (Eapen et al., 2014) to Hungarian and applied it in Hungary with the approval of the authors. This instrument measures the parental quality of life of autistic children and adults. It has 48 items divided into two subscales one of which (A) measures general quality of life, while the other (B) is designed to ascertain the impact of specific ASD features. Within the AS group, parental quality of life indices exhibited strong correlations with one another, and with the QOLA scales as well (shown in the table *Correlations between the quality of life indices in the two groups, Online Resource 1*). The same result was observed in the NT group with one exception: QOLA-B, the impact of ASD symptoms on quality of life was unrelated to the other quality of life domains.

### Socio-Demographic and Economic Factors

Data were collected about participating parents’ gender, age, family status, education, type of residence (region and area of residence type), estimated relative income, physical and mental illnesses (if any), and their own ASD traits (if any). Basic information concerning all individuals being raised in the family was also ascertained (number; gender; age; strengths, possible disorders, and illnesses).

### Parental Psychological Well-Being

The *Depression, Anxiety and Stress Scales (DASS)* was used to collect information about parental negative emotional status (Lovibond & Lovibond, 1995; Crawford & Henry, 2003). This questionnaire consists of 42 statements describing negative emotional states, and the individual is asked to rate, on a four-point (frequency/severity) scale, the occurrences of these types of emotional states over the past week (Lovibond & Lovibond, 1995; Crawford & Henry, 2003). Sense of coherence was defined with orderliness (or being well-organized) and having a meaning to one’s life; the *Sense of Coherence Scale* assesses how people view life and identifies how they use their resistance resources to maintain and develop their health (Antonovsky, 1987). The original instrument comprises 29 items, we used a short, 13-item version available in Hungarian (Antonovsky, 1987, 1993; Balajti et al., 2007).

The *Parenting Sense of Competence Scale* (Johnston & Mash, 1989) is a tool for measuring parents’ satisfaction, and parenting efficacy. This questionnaire has 17 items and it has been adapted in a Hungarian version (Ribiczey et al., 2016). The aim is to obtain (i) a picture of the individual’s feelings related to parental role (parental satisfaction), and (ii) a subjective assessment of their ability to meet the requirements related to parental role – or one’s perceived parental competence (parenting efficacy) (Ribiczey et al., 2016). The Hungarian adaptation of the *Caldwell Social Support Questionnaire* (Kopp & Kovács, 2006) by our group served as the basis for compiling a list of 12 potential supporters (e.g., neighbor; spouse; church group), to rely on in difficult situations. The level of support available from each of these sources can be indicated on a 1 to 4 scale (can’t rely on them at all – can rely on them very much). We also asked parents whether they needed professional support, and whether it was available to them if they did.

### Individual Characteristics of Participants’ Children

Individuals’ gender, age, and intellectual level was recorded based on parental report. Information was also collected about the type of care that the autistic/neurotypical individuals received: the institution where they studied or worked, and the types of support (autism-specific or other) they received within or outside that institution. Parents indicated their satisfaction with their child’s current progress, and education on a 0 to 10 scale (0 meaning maximal dissatisfaction; 10 meaning maximal satisfaction). In the AS group, age of the first ASD diagnosis was also recorded; in addition, parents in both groups supplied information about comorbidities. Severity of ASD was established using the *Social Communication Questionnaire (SCQ)* completed by the parents (Rutter et al., 2003). It surveys repetitive and stereo-typed behaviours and socio-communicative symptoms. In the present study, the current form was used, in which parents reported their child’s symptoms in the past 3 months (Rutter et al., 2003). Parents’ experience about their child’s *strengths, talents*, and outstanding performance was examined in 13 areas (plus an open ”other” option). The questions were constructed using the Autism Diagnostic Interview, Revised (ADI-R) items on strengths (Lord et al., 1994). A summary score was calculated from the indicated strengths and outstanding abilities. Fifteen statements related to challenging behaviours, each of which was rated by parents on a three-point scale indicating frequency (never; rarely; often). These statements were formulated relying on frequently used, standardized instrument: Vineland Adaptive Behaviour Scale 3rd Edition (VABS-3) (Sparrow et al., 2016), ADI-R (Lord et al., 1994).

### Data Analysis

First, we compared the scores on the quality-of-life indices in the two groups including (1) between-group comparisons of the levels, and (2) group-specific correlation matrices for the six indices. Second, we also compared our groups in terms of psychological well-being. Three groups of variables were used in this comparison (for background principles see above in Conceptual framework): (1) Parental socio-economic and demographic status: gender, age, education, area of residence type, estimated relative income, number of children; (2) parental well-being: symptoms of depression, anxiety, stress, sense of coherence, parental sense of competence, social support, professional support for the parent; (3) characteristics of participants’ children: gender, age, SCQ, number and intensity of types of challenging behaviour, number of strengths, IQ, parental satisfaction with child’s education. Third, we conducted multiple regression analyses to obtain models of how psychological well-being factors predict the outcomes of quality of life scales.

## Results

### Differences in Quality of Life Between AS and NT Groups

We found significant differences accompanied by substantial effect sizes in parental quality of life in all four domains of the WHOQOL-BREF: physical health (BREF1) (Z=-7.33, *p* < 0.001), psychological domain (BREF2) (Z=-8.36, *p* < 0.001), social relationships (BREF3) (Z=-9.02, *p* < 0.001), environment domain (BREF4) (Z=-8.63, *p* < 0.001), and the two scales of the QOLA: QOLA-A general domain (Z=-10.61, *p* < 0.001), specific ASD features on quality of life QOLA-B (Z=-10.74, *p* < 0.001) between the two groups (AS and NT); parents in the NT group had higher scores (Table 3).

**Table 3:** Differences in parental quality of life between the two groups

	Group	Mean	Welch test (W) Cohen’s d (d)	Yuen-test (20% trimming) (Y) Trimmed Cohen’s d (d)	Mann-Whitney test (Z)	Sig.	CL
BREF1 (Phys WB)	AS	69.72	W(803.5)=-8.086 Mean diff.:-7.98 CI95=[-9.919;-6.049] d=-0.540	Y(489.3)=-8.067 T mean diff.:-8.22 CI95=[-10.21;-6.22] d=-0.890	-7.33	< 0.001	0.64
	NT	77.7					
BREF2 (Psych WB)	AS	58.90	W(808.3)=-8.636 Mean diff.:-9.06 CI95=[-11.11;-7.00] d=-0.575	Y(480.1)=-7.954 T mean diff.:-9.53 CI95=[-11.88;--7.18] d=-0.886	-8.36	< 0.001	0.66
	NT	67.96					
BREF3 (Social WB)	AS	53.99	W(754.6)=-9.864 Mean diff.:-12.77 CI95=[-15.3;-10.23] d=-0.677	Y(503.4)=-8.223 T mean diff.:-12.36 CI95=[-15.30;-9.41] d=-0.882	-9.02	< 0.001	0.68
	NT	66.76					
BREF4 (Envir WB)	AS	57.52	W(798.7)=-9.296 Mean diff.:-9.6 CI95=[-11.62;-7.75] d=-0.623	Y(486.0)=-9.763 T mean diff.:-9.9 CI95=[-11.89;-7.91] d=-1.081	-8.63	< 0.001	0.67
	NT	67.12					
QOLA -A	AS	93.88	W(803.3)=-11.802 Mean diff.:-0.129 CI95=[-0.150;-0.107] d=-0.788	Y(498.0)=-11.698 T mean diff.:-0.135 CI95=[-0.158;-0.113] d=-1.255	-10.61	< 0.001	0.71
	NT	108.28					
QOLA -B	AS	48.86	W(639.4) = 11.099 Mean diff.:0.165 CI95=[0.136;0.194] d = 0.807	Y(301.3) = 9.394 T mean diff.:0.191 CI95=[0.151;0.231] d = 1.221	-10.74	< 0.001	0.64
	NT	35.64					

Results of the Welch test are presented as standard deviations were unequal; the Yuen test with 20% trimming was used because normality was violated within the groups. The Mann-Whitney test was calculated because questionnaire scores may need to be considered ordinal scales. All these tests indicate a substantial between-group difference for all indices. Column CL is the common language effect size for the Mann-Whitney test (McGraw & Wong, 1992)

Below we examine the system of factors influencing parental quality of life in the two groups, for which Table 4 provides support.

**Table 4:** Road map of correlated factors with QOL

Quality of Life (QOL)			
WHO-BREF1		Physical domain	
WHO-BREF2		Psychological domain	
WHO-BREF3		Social domain	
WHO-BREF4		Environmental domain	
QOLA-A		General quality of life	
QOLA-B		Autism related quality of life	
**Socio-demographic and economic factors (SD)**	**Parental psychological** **well-being (PWB)**		**Individual characteristics** **(child)**
Parental gender	Parental competence (PSOC)		Child’s gender
Parental age	Coherence (SOC)		Child’s age
Area of residence type	DASS-depression		Child’s strengths
Parental education	DASS-anxiety		Child’s symptoms severity
Estimated relative income	DAS-stress		Number and intensity of challenging behaviour
Number of children	Social support (informal)		IQ
	Professional support (formal)		Parental satisfaction with child’s education

### Socio-Demographic and Economic Factors

Parental gender had a decisive influence on quality of life. In the AS group, physical health (BREF1), had a mean of 77 ± 13 for fathers, and 68 ± 26 for mothers, which was significant (Yuen test: Y = 4.804, *p* < 0.001 [after Bonferroni correctionfor 6 repeated tests] trimmed Cohen’s d = 0.790). In the psycological domain (BREF2), fathers’ mean was 67 ± 15 and mothers’mean was 57 ± 17 which was again significant (Y = 5.571, *p* < 0.001 [Bonferroni: 6 tests] trimmed Cohen’s d = 1.050). Regarding social relationships (BREF3) the mean scores were: mothers 53 ± 20, fathers: 57 ± 18 (Y = 1.699, *p* = 0.116 trimmed Cohen d = 0.282). Finally, for the environment domain (BREF4), the difference again indicated a life quality advantage for fathers: mothers’ mean/sd = 56 ± 17, fathers’ mean/sd = 65 ± 15 (Y = 4.267, *p* = 0.0012 [Bonferroni-corrected for 6 tests] trimmed Cohen d = 0.925). In the general quality of life scale QOLA-A subscale, fathers’ mean/sd was 103 ± 18, whereas mothers scored 92 ± 20 which again indicated a better quality of lifes for fathers (Y = 4.368, *p* < 0.001 [Bonferroni correction for 6 tests] trimmed Cohen’s d = 0.871). The subscale measuring the influence of specific ASD features on quality of life (QOLA-B) found that the parent groups did not differ (fathers: 44 ± 18; mothers: 50 ± 16; Y=-2.839, *p* = 0.0744 trimmed Cohen’s d=-0.688, although the tendency-level difference pointed in the same direction as on the other subscales). Comparing the two parent groups in the NT sample, none of these six quality-of-life indicators showed a significant difference.

Next, we examined the correlations between (1) the six quality of life indicators, and (2) the following socio-demographic variables: parental age, area of residence type (farmstead; village; township; county seat; capital) education (elementary school or vocational high school without baccalaureat; high school with baccalaureat; higher education [college or university]); estimated relative monthly income (five categories: very low; low; average; moderately above average; substantially above average), and number of children. Three types of correlation coefficient were calculated in each case (Pearson; Wilcox r_pb_, and Kendall tau), however, only Kendall tau was interpreted in the case of education, area of residence type, and estimated relative income, as these variables are ordinal. The five general quality of life scales were found to be significantly correlated with income in both groups^1^ (except BREF3 in the NT group). Magnitudes of the correlation coefficients were between 0.177 and 0.556 (the value for BREF3 in NT was 0.137). QOLA-B did not correlate with income. In addition, BREF4 correlated with Education (tau: 0.212 [AS], 0.175 [NT]); so did QOLA-A, but only in the AS group (tau = 0.140). Despite statistical significance, most of these correlations are moderate or low, therefore they should be interpreted with caution (shown in the table *Correlations between quality of life indicators and socio-demographic variables, Online Resource 2*).

### Parental Psychological Well-Being

Stronger correlations were found between measures of psychological well-being (SOC, DASS, PSOC, social and profession support), and quality-of-life scales. This finding is easy to interpret as psychological well-being is a key factor in one’s quality of life (shown in the table *Correlations between parental psychological factors of well-being, and the quality of life scales, Online Resource 3*).

### Individual Characteristics of Participants’ Children

Regarding the variables characterizing individuals, we found that the frequency of challenging behaviours negatively influenced quality of life in both groups; this was indicated by all of the six scales used. Again, the only exception was QOLA-B in the NT group. We also found that SCQ values significantly correlated with the four BREF scales and QOLA-A in the AS, but not in the NT group. QOLA-B showed a significant positive correlation with SCQ in both groups. Parents’ satisfaction with their child’s education was also positively related to BREF4, QOLA-A, and QOLA-B, but only in the AS group (shown in the table *Correlations between parents’ quality of life measured by the six scales, and characteristics of participants’ children including severity of symptoms, Online Resource 4*). We also investigated whether the QOL of each subgroup in the AS group varied with the age (early years/school age/adult) of the individual. A two-way mixed ANOVA (Age group (3) X quality of life scale (6)) with follow-up analysis indicated that, of the six QOL scales, only BREF1 and QOLA-B scores distinguished the three age groups significantly, however, effect sizes (η^2^) were small even in these cases (see details in *Comparison of the age subgroups of the AS group in terms of quality of life scores, Online Resource 5*).

### Multiple Linear Regression Model-Stepwise (Quality of Life in the AS and NT Groups)

Summarizing so far, we found a correlation between parental quality of life and indices of (1) parental well-being, (2) characteristics of their children and (3) socio-economic status, in both groups. We examined which of the factors determining quality of life are specific to parents of autistic children/adults; in other words, how do the relevant factors differ in the two groups? Quality of life was measured by the six scales described above: QOLA-A, QOLA-B and the four BREF domains. These indices served as dependent variables of twelve multiple regression analyses, as for each of the six dependents, the two groups were tested separately. The predictors in these analyses were indentical in the two groups (initially date of the first diagnosis was included as a predictor in the AS group, but subsequently it was omitted as it was not significant). In each model the AS group exhibited the same or a larger number of significant predictors than the NT group. We started with 20 predictor variables thematically divided in three groups, although these groups were not reflected in the model, as we used stepwise entry.

- Parental socio-economic and demographic status (6): gender, age, education, area of residence type, estimated relative income, number of children.

- parental well-being (7): symptoms of depression, anxiety, stress, sense of coherence, parental sense of competence, social support, professional support for the parent;

- individual characteristics (7): gender, age, SCQ, number and intensity of types of challenging behaviour, number of strengths, IQ, parental satisfaction with child’s education.

**Fig. 1:**
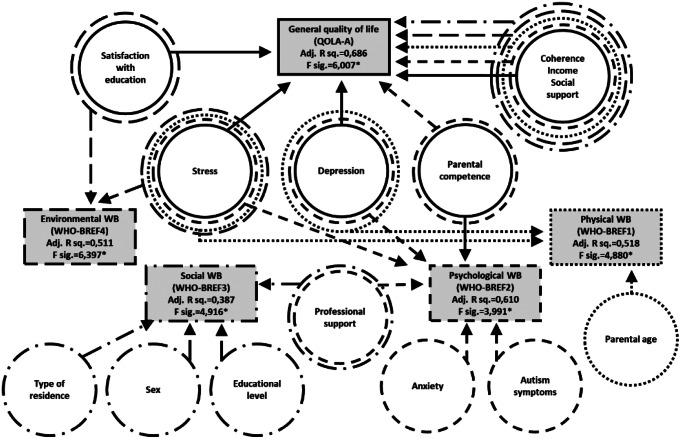
Regression model – predictors of general quality of life in AS group

**Fig. 2:**
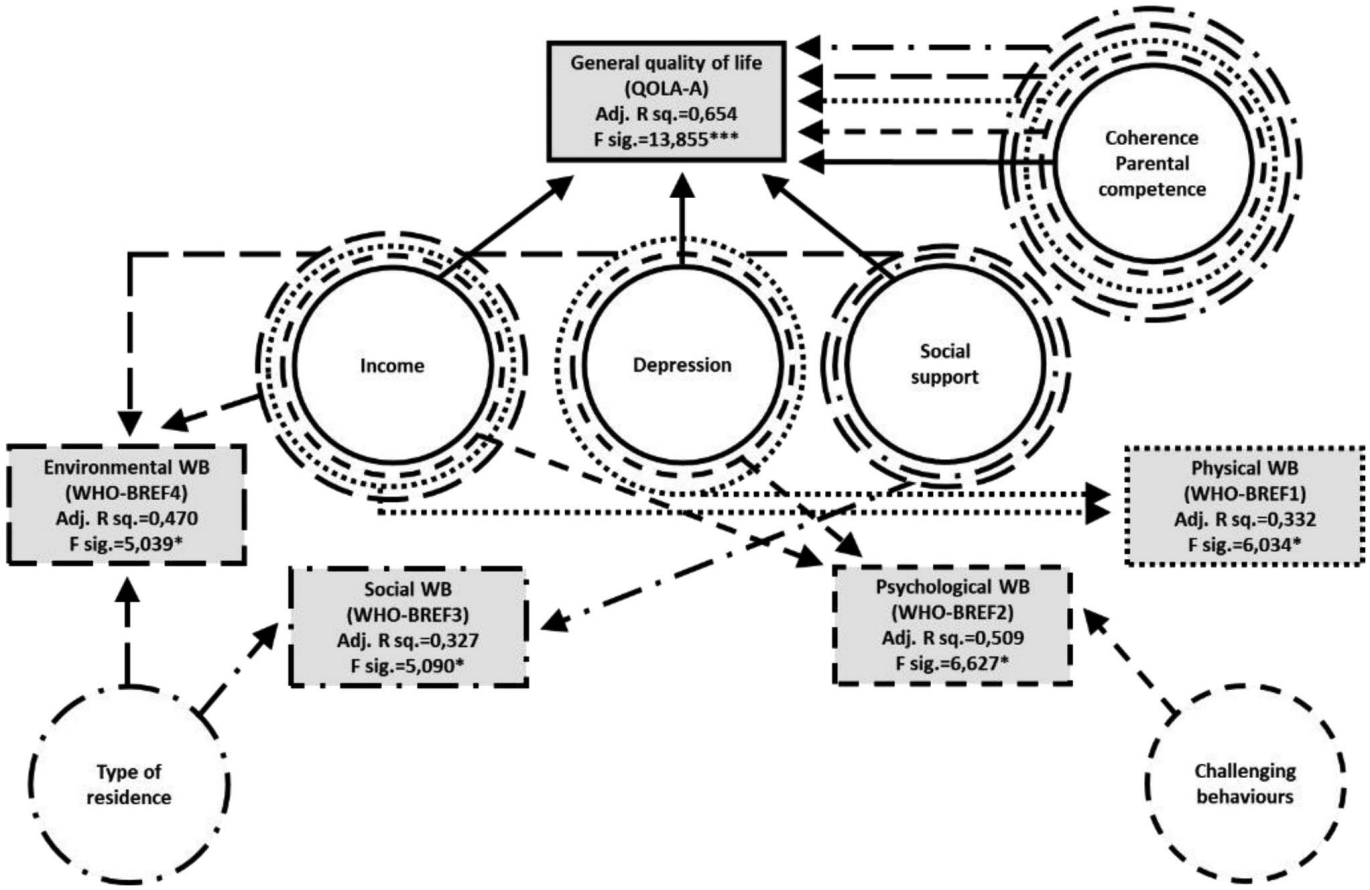
Regression model – predictors of general quality of life in NT group

Figures 1 and 2 show the regression model of predictors of general quality of life in AS and NT groups. (Details of the regression models are shown in tables of *Online Resource 6*).

Regarding the predictors of *QOLA-A*, five variables proved significant in both groups: (G1)^2^ sense of coherence, (G2) satisfaction with family income, (G3) symptoms of depression, (G4) social support, and (G5) parental sense of competence. In the AS group, two more predictors were identified: (A1)^2^ stress, and (A2) parent satisfaction with child’s education. No group-specific predictors were identified in the NT group. Overall model strength (in terms of adjusted R squares) was comparable in the two groups.

*QOLA-B*, the measure of effect of ASD symptoms on parental quality of life was significantly predicted by the strength of child symptoms (SCQ score) in the AS group (A1/G1). Two further, group-specific predictors were found in the AS group: (A1) number and intensity of different types of challenging behaviours, (A2) symptoms of depression. In the NT group the overall strength of the model was much lower, with the only significant additional predictor being (N1)^2^ parental age (higher age correlated with poorer quality of life).

The *BREF-1 (Psysical domain of quality of life)* index had three significant predictors in both groups (that also figured in the QOLA-A subscale): (G1) sense of coherence, (G2) satisfaction with family income, (G3) symptoms of depression. In addition to these, in the AS group (A1) parental age, (A2) stress, and (A3) social support were also significant, whereas in the NT group, (N1) parental sense of competence joined to G1-G3.

*BREF-2 (Psychological domain of quality of life)* had an overlapping, but not identical set of predictors in the two groups. Symptoms of depression, (G1) sense of coherence, (G2) satisfaction with family income, and (G3) parental sense of competence were significant in both groups. In addition, we found five predictors specific to the AS group: (A1) social support, (A2) anxiety, (A3) stress, (A4) professional support, and (A5) SCQ score. In the NT group the only specific predictor was (N1) challenging behaviours.

For the *BREF-3 (Social domain of quality of life)* general predictors were: (G1) sense of coherence, (G2) social support, (G3) area of residence type (area of residence types with larger population were associated with poorer quality of social life). Group-specific predictors in AS were: (A1) parental level of education, (A2) satisfaction with family income, (A3) professional support, (A4) gender. Parental sense of competence (N1) was the only NT-specific predictor.

In *BREF-4 (Environmental domain of quality of life)* the two groups shared (G1) parental sense of competence, (G2) social support, (G3) sense of coherence as predictors. In the AS group, specific predictors were (A1) stress, and (A2) satisfaction with child’s education. In NT, (N1) parental sense of competence, and (N2) area of residence type proved significant.

## Discussion

To the best of our knowledge this is the first systematic large-sample study examining quality of life of parents of individuals living with ASD in Central-Eastern Europe. The present study examined the quality of life and psychological well-being of parents of over 500 autistic, and over 300 neurotypical individuals using stratified sampling. We presented results of the first comprehensive study addressing quality of life of parents of autistic individuals in Hungary. However, our data affords further analyses that could provide an a more detailed picture of the topic. A number of factors known to affect parental quality of life (such as socio-demographic and socio-economic characteristics, the autistic/neurotypical individuals’ characteristics, and information about intervention) were included as indepdenent variables. Since studies from other countries have repeatedly indicated that the quality of life, and psychological well-being of parents of neurotypical children is better than that of parents raising autistic children (Vasilopoulou & Nisbet, 2016; Wang et al., 2018), our main objective was to uncover the underlying factors in Hungary. Participants came from a broad age range. 11% of individuals in the AS group were in the range of borderline intellectual functioning, whereas 57% were in the average or above-average range. These ratios approximate international frequency data fairly well; in studies of the Autism and Developmental Disabilities Monitoring (ADDM) Network (conducted in 2016; published in 2020) 24% of autistic participants fell in the borderline range, whereas 42% had average or higher intellectual ability (Maenner et al., 2020). Our results are consistent with previous findings from other countries that parents of autistic individuals showed significantly lower QOL in all domains of general and ASD related QOL (Markowitz et al., 2016; Pisula & Porębowicz-Dörsmann, 2017).

### Quality of Life; Socio-Demographic, and Economic Factors

Domains of the two instruments measuring parental quality of life were strongly correlated within and between instruments in both groups. Moreover, In the NT group we found no association between parental gender and quality of life, whereas in the AS group mothers reported lower scores in certain domains including general quality of life, physical, psychological, and environmental well-being. Mothers and fathers did not differ in the social dimension of WHOQOL-BREF, nor did they do so in terms of the effects of ASD symptoms on quality of life (QOLA-B). Quality of life of mothers and fathers, and their determining factors have been previously investigated in recent years (Mugno et al., 2007; Dardas & Ahmad, 2014; Eapen & Guan, 2016; Wang et al., 2018; Eapen et al., 2022). Although previous studies have indicated that mothers’ quality of life is lower than that of fathers in most domains, (Dardas & Ahmad, 2014; Vasilopoulou & Nisbet, 2016; Mathew et al., 2019), the reasons for this difference are as yet unclear. Studies involving both fathers and mothers have observed that fathers’ personal, parental, and familial stress correlated negatively with quality of life, especially on mental and physical health domains (Dardas & Ahmad, 2014; Eapen & Guan, 2016). In our sample neither parental age, nor area of residence type was related to parental quality of life. In the AS group (but not in the NT group), level of parental education was significantly correlated with WHOQOL-BREF’s environmental domain: the higher the parental educational level, the better their access to high-quality dwelling, security, and services including leisure (Derguy et al., 2018). Also in line with findings from other countries (Dardas & Ahmad, 2014; Čolić et al., 2019), financial characteristics were particularly strongly correlated with quality of life in both groups and, with the exception of QOLA-B, parents with higher incomes reported better quality of life in all domains. In contrast to previous findings (Dardas & Ahmad, 2014; Vasilopoulou & Nisbet, 2016), we did not find a correlation between the number of siblings (i.e. the number of children in the family) and quality of life in either group.

### Parental Psychological Well-Being, and its Relation to Quality of Life Measures

In the present study, each of the six measures of parental quality of life correlated well with each dimension of psychological well-being in the AS group, the only exception being QOLA-B and social support. Those parents of autistic individuals who had a better sense of coherence, parental competence, social support, and availability of professional support, while having less stress, anxiety, and depressive symptoms, tended to report better quality of life. Similar pattern was found in the NT group as well, however, here the level of formal and informal support was not correlated with all quality of life domains. Parental quality of life can be improved by involving parents in their child’s care supported by specific parent support programmes (Lichtlé et al., 2019; Musetti et al., 2021; Turnage & Conner, 2022).

### Quality of Life and Characteristics of Participants’ Children

In neither group did individuals’ age correlate with parental quality of life. Nor did individuals’ strengths as potential protective factors correlate with quality of life, in either group. We similarly failed to find a relationship between individuals’ intellectual ability and parental quality of life, which is in accordance with earlier findings (Peters-Scheffer et al., 2012; Vernhet et al., 2022; Eapen et al., 2022). Next, parental satisfaction with child’s education showed only a marginally significant correlation with quality of life, even though this relationship has been confirmed in previous studies (Frantzen et al., 2016; Hodgetts et al., 2017). We did find, however, that challenging behaviours resulted in increase in parental stress, and poorer quality of life, as has been shown in earlier literature reviews (Eapen & Guan, 2016; Enea & Rusu, 2020). In the AS group, this factor was related to lower parental quality of life in all domains, whereas in the NT group it was negatively related to general quality of life, and the physical, social, and environmental QOL domains. Stronger symptoms (higher SCQ score) were accompanied by lower general, physical, psychological, and ASD-symptom-related quality of life in the AS group (Cappe et al., 2018). In the NT group this relationship was confirmed only for QOLA-B.

### Multiple Regression Models of Quality of Life in the Two Groups

Of the entered 20 variables, four did not prove to be significant predictors for any QOL domain, in either group: number of siblings; child’s gender, age, and intellectual ability. Altogether in the six models, the dominant predictors were parental psychological well-being, and estimated relative income, which are not autism-specific factors.

Comparing models of the two groups, differences were found in these two predictors; in addition, parental satisfaction with child’s education in the AS group, and parental sense of competence in the NT group were significant predictors.

In our sample, parental quality of life was strongly predicted by sense of coherence, estimated relative income, strength of depressive symptoms, and social support. In the neurotypical group, parental sense of competence was another important predictor for a number of QOL domains, whereas in the AS group a greater variation of determining factors were found. In a previous study by Ilias et al. (2018), parental stress was found to have an influence on general quality of life, namely the physical, psychological, and environmental domains, and this study also emphasized the role of social support as a stress-relieving factor for parents with autistic children; the latter effect was also observed in the present study. Further stress-related factors examined by these authors (Ilias et al., 2018) were severity of ASD symptoms, financial difficulties, parents’ perception and understanding toward autism, and parents’ anxiety and worries about their child’s future – the latter two were not assessed in the present study.

It is also important to note that both social (Hsiao, 2016; Vasilopoulou & Nisbet, 2016), and professional support tended to improve quality of life (psychological and social domains) in the AS group, in our study as well as in previous studies (Musetti et al., 2021; Wang et al., 2022).

General quality of life, and the environmental domain, of parents of autistic individuals was found to be directly influenced by satisfaction with child’s education, in earlier research (Derguy et al., 2018) and this was replicated in the present study. Further, parents of autistic children burnout has been correlated with their child’s social behaviour and parental quality of life in that parents having higher levels of burnout experience exhibiting lower quality of life as a direct impact of their child’s social behaviour (Wang et al., 2018).

In our sample, autism-related aspects of quality of life were observed to be influenced by the frequency and intensity of the individual’s challenging behaviours, strength of the individual’s ASD symptoms, and the parents’ susceptibility to depression. This is in keeping with other studies that have found an association between better parental quality of life and lower severity of the child’s ASD features and better adaptive functioning (Eapen et al., 2022).

These results are the first comprehensive interpretations of our findings. However further analyses is indicated to provide an accurate picture of the quality of life of parents of Hungarian autistic individuals. From international comparative studies, we see that our results are in line with the cultures of other Western countries, with only slight differences (van Kessel et al., 2020; Eapen et al., 2023). Hungary is part of the European Union, and there are no significant cultural, ethnic and religious differences between it and other EU countries, therefore our results may be valuable for other countries. However our country’s unique political situation and values, the unpredictable and uncertain future in Hungary and the heavy financial burden also cause difficulties for families (NT) in general (EUROSTAT, 2023). More over it is seen and known that these affect families supporting an autistic individual even more and have an even greater impact on families well-being and quality of life (AS) (Rothwell et al., 2019; Zhao et al., 2023).

### Limitations

In this article we have presented results of a large-sample study on parental quality of life in the context of autism, which have important practical implications; however some limitations need highlighting as well. Unfortunately, not all tools used in Hungarian language have a validated, culturally adapted version, so DASS (Szabó, 2010), SCQ and QOLA are still to come. The under-representation of parents with low level education in the sample raises some questions about the generalizability of the findings: (1) despite our efforts we were not able to recruit underprivileged families – hence their quality of life remains to be explored (2) we do not know whether our efforts toward paper-based data collection were insufficient, or ASD is under-diagnosed in low-education groups. We consider it another limitation that most parents in the sample completed the questionnaires online, therefore we had no control over the circumstances, nor could we verify the obtained data. In particular, the child’s diagnosis, including their IQ was also collected through parental report. In the case of autism spectrum disorder, further sophisticated analysis of known factors affecting the quality of life of parents (e.g. time since first ASD diagnosis, or other characteristics of children) is needed in different AS subgroups. Further, the data presented here are quantitative, and even though we contacted a substantial portion of Hungarian families with autistic children and adults, the qualitative data we collected about quality of life is not included in this study.

### Implications for Practice

Personal characteristics, and environmental factors very strongly influence the well-being, and quality of life of parents with individuals on the autism spectrum. Based on our findings and considering an ecological approach, a comprehensive model of intervention and supports including the family is essential for promoting the overall wellbeing and quality of life of those impacted. Our study confirms that attention to the parents’ social situation, and providing the best possible care for their child are important tasks for professionals. Further challenges ensue from families’ additional psychosocial (e.g., financial) needs. In additon to intervention directed primarily for the child, an important goal is to support parents in their role and provide health and social care including parent training programs that focus on the quality of life (Lichtlé et al., 2019).

## Footnotes

1 After Bonferroni correction for 30 repetitions.

2 *G = general predictor, found in both groups, „A”=autism-related predictor, „N”= predictor found only in neurotypical group.
